# Comparative Analysis of Microbial Species and Multidrug Resistance Patterns in Acute Cholangitis Patients with Cholecystectomy: A Single-Center Study

**DOI:** 10.3390/diseases12010019

**Published:** 2024-01-06

**Authors:** Bogdan Miutescu, Deiana Vuletici, Calin Burciu, Felix Bende, Iulia Ratiu, Tudor Moga, Eyad Gadour, Shruta Reddy, Vasile Sandru, Gheorghe Balan, Greta Dancu, Felix-Mihai Maralescu, Alina Popescu

**Affiliations:** 1 Department of Gastroenterology and Hepatology, “Victor Babes” University of Medicine and Pharmacy Timisoara, Eftimie Murgu Square 2, 300041 Timisoara, Romania; miutescu.bogdan@umft.ro (B.M.); calin.burciu@umft.ro (C.B.); bendefelix@gmail.com (F.B.); ratiu_iulia@yahoo.com (I.R.); moga.tudor@gmail.com (T.M.); greta.dancu@umft.ro (G.D.); popescu.alina@umft.ro (A.P.); 2 Advanced Regional Research Center in Gastroenterology and Hepatology, “Victor Babes” University of Medicine and Pharmacy, 300041 Timisoara, Romania; 3 Department of Gastroenterology, Faculty of Medicine, Pharmacy and Dental Medicine, “Vasile Goldis” West University of Arad, 310414 Arad, Romania; 4 Department of Gastroenterology, King Abdulaziz Hospital-National Guard Health Affairs, Al Ahsa 31982, Saudi Arabia; eyadgadour@doctors.org.uk; 5 Department of Medicine, Zamzam University College, Khartoum 11113, Sudan; 6 Department of General Medicine, SVS Medical College, Yenugonda, Mahbubnagar 509001, Telangana, India; shrutareddy097@gmail.com; 7 Department of Gastroenterology, Clinical Emergency Hospital of Bucharest, 105402 Bucharest, Romania; drsandruvasile@gmail.com; 8 Department 5, “Carol Davila” University of Medicine and Pharmacy, 050474 Bucharest, Romania; 9 Department of Gastroenterology, “Grigore T. Popa” University of Medicine and Pharmacy, 700115 Iași, Romania; balan.gheo@me.com; 10 Division of Nephrology, Department of Internal Medicine II, “Victor Babes” University of Medicine and Pharmacy Timisoara, Eftimie Murgu Square 2, 300041 Timisoara, Romania; mihai.maralescu@umft.ro; 11 Centre for Molecular Research in Nephrology and Vascular Disease, “Victor Babes” University of Medicine and Pharmacy Timisoara, Eftimie Murgu Square 2, 300041 Timisoara, Romania

**Keywords:** acute cholangitis, cholecystectomy, antimicrobial resistance

## Abstract

This study aimed to compare microbial species and multidrug resistance patterns in acute cholangitis patients with and without a history of cholecystectomy, highlighting potential differences We hypothesized that post-cholecystectomy patients would exhibit distinct microbial spectra and resistance patterns. Conducted at a western Romanian hospital specializing in gastroenterology and hepatobiliary diseases from 2020 to 2023, this retrospective study included 488 acute cholangitis patients, divided into groups based on their cholecystectomy history. Bile and blood samples were analyzed for microbial identification and antibiotic susceptibility using VITEK^®^2. Positive biliary cultures were found in 66% of patients. The cholecystectomy group showed a higher prevalence of multidrug-resistant organisms, with 74.4% exhibiting resistance compared to 31.5% in the non-cholecystectomy group (*p* < 0.001). Notable microbial differences included higher occurrences of *Escherichia coli* (40.2%) and *Enterococcus* spp. (32.4%) in the cholecystectomy group. Resistance to Piperacillin/Tazobactam and Penems was significantly higher in this group, with odds ratios of 3.25 (*p* < 0.001) and 2.80 (*p* = 0.001), respectively, for the development of multidrug-resistant (MDR) bacterial species. The study confirmed our hypothesis, revealing distinct microbial profiles and a higher prevalence of multidrug resistance in acute cholangitis post-cholecystectomy patients. These findings underscore the need for tailored antibiotic strategies in managing acute cholangitis in this patient demographic.

## 1. Introduction

Acute cholangitis has been increasingly associated with multidrug-resistant (MDR) organisms, complicating treatment strategies [1]. The prevalence of acute cholangitis has seen an upward trend, with studies indicating an increase in hospitalizations for cholangitis in the United States between 2005 and 2014, reflecting a growing burden of gallstone disease and associated complications [2,3,4]. This rise is paralleled by a growing concern over the emergence of MDR pathogens, which pose a significant threat to patient outcomes [5,6].

The microbial spectrum in acute cholangitis has evolved over the years. Initially dominated by *Escherichia coli* and *Klebsiella pneumoniae*, recent studies have reported a diversification in the microbial species involved, including the rise of anaerobic bacteria [7,8,9]. This shift in the microbial landscape has important implications for antibiotic therapy, as the efficacy of traditional regimens is increasingly being questioned [10]. The role of cholecystectomy, a common treatment for gallstone-related diseases, in altering microbial patterns and resistance profiles in acute cholangitis patients remains underexplored [11].

Multidrug resistance in acute cholangitis is a growing concern, with studies reporting resistance rates as high as 30–40% in certain regions [12]. The mechanisms of resistance are varied, including extended-spectrum beta-lactamases (ESBLs) and carbapenemases, which significantly limit treatment options [13,14]. The clinical impact of these MDR organisms is profound, often leading to prolonged hospital stays, increased healthcare costs, and higher mortality rates [15,16]. The relationship between cholecystectomy and the development of MDR in cholangitis patients is a critical area of investigation, with potential implications for both surgical and pharmacological management.

Evidence suggests that patient outcomes in acute cholangitis are heavily influenced by the timely administration of appropriate antimicrobial therapy [17]. However, the rise of MDR organisms has made the selection of effective antibiotics increasingly complex [18]. The need for empirical therapy that covers a broad range of potential pathogens, while also considering local resistance patterns, is essential [19]. This challenge is further compounded in post-cholecystectomy patients, where alterations in bile flow and gut microbiota may affect the susceptibility and presence of various microbial species [20].

Recent advances in microbial sequencing and resistance pattern analysis offer new opportunities to understand the complex interplay between microbial species and drug resistance in acute cholangitis [21]. These technologies hold promise for the development of more targeted and effective treatment strategies, particularly in the context of post-cholecystectomy patients [22,23,24]. However, there remains a significant gap in comprehensive studies that integrate microbial species analysis with multidrug resistance patterns in this patient group.

Given the lack of published data on acute cholangitis in the healthcare setting of Romania, our study aims to fill this significant gap by analyzing the specific microbial spectrum and multidrug resistance patterns prevalent in the Romanian population. This approach is critical, as local bacterial profiles and resistance trends can markedly differ from global patterns, and are in constant change, necessitating an ongoing data report, and different strategies for effective management and treatment of acute cholangitis, particularly in the context of post-cholecystectomy care.

In this study, we hypothesize that acute cholangitis in post-cholecystectomy patients is characterized by a unique microbial spectrum and distinct multidrug resistance patterns compared to non-cholecystectomy patients. Our aim is to conduct a comparative analysis of microbial species and multidrug resistance patterns in these two patient groups, providing insights that could inform more effective management strategies for acute cholangitis in the context of cholecystectomy. 

## 2. Materials and Methods

### 2.1. Research Framework and Ethical Considerations

This retrospective study was conducted at a major regional hospital in western Romania, specializing in gastroenterology and hepatobiliary diseases. The study period spanned from 2020 to 2023, focusing on patients diagnosed with acute cholangitis who had a history of cholecystectomy. Ethical approval was obtained from the hospital’s Institutional Review Board, in alignment with the principles of the Declaration of Helsinki. Confidentiality and privacy of patient data were strictly maintained. Informed consent was obtained from all participants before data collection.

Patients in both the case and control groups had a confirmed diagnosis of acute cholangitis as per the Tokyo guidelines and were treated with antibiotics, according to standard of care. The control group included patients with a history of cholecystectomy, chosen to compare the impact of gallbladder removal and biliary tree manipulation on microbial spectrum and resistance patterns. Bile samples were collected from all patients as a necessary procedure in the diagnosis and management of acute cholangitis, providing data on the bacterial profile and antibiotic sensitivity testing.

### 2.2. Participant Selection and Sample Collection

For this study, acute cholangitis (AC) was diagnosed by adhering to the criteria outlined in the latest clinical guidelines (Tokyo Guidelines TG18) [25]. Enrolled participants were categorized into two groups: Group A (patients with a history of cholecystectomy) and Group B (patients without a history of cholecystectomy), identified at their initial hospital admission. Those with a history of AC post-endoscopic retrograde cholangiopancreatography (ERCP) or currently on antibiotics for other conditions were excluded. Patients were also excluded from the study if they refused to share their data, if they lacked the ability to consent, and had missing medical information. 

Following the diagnosis of acute cholangitis, all participants received antibiotic therapy tailored to their specific classification of AC severity, in accordance with the latest guidelines. The treatment regimens varied from ampicillin/sulbactam, ciprofloxacin, or levofloxacin for milder cases, to more potent antibiotics such as ceftriaxone or meropenem for severe instances. Bile and blood samples were collected for microbial analysis. 

Upon hospitalization for moderate to severe acute cholangitis, in accordance with the TG18 recommendations, blood cultures were promptly initiated. Bile samples were collected after cannulation using a sphincterotome, prior to initiating any therapeutic intervention. To minimize contamination, the first 5 mL of bile was discarded, and the subsequent 5 mL was collected into a sterile container designed for both anaerobic and aerobic bacterial culturing. These specimens were then incubated at 37 °C for at least seven days or until microbial growth was detected. The VITEK^®^ 2 system (bioMérieux, Marcy-l′Étoile, France) was employed to determine the antibiotic susceptibility, specifically the minimum inhibitory concentration (MIC), of the isolates. The results were interpreted in line with current clinical guidelines [26]. Antibiotic resistance and susceptibility were established based on the standards and criteria set forth by the Clinical and Laboratory Standards Institute (CLSI) for all bacteria cultured [27].

Additional diagnostic measures, such as B-mode sonography and endoscopic ultrasound (EUS), were employed to determine the nature of biliary obstruction. ERCP, conducted with high-precision instruments, was used both for diagnostic and therapeutic purposes. Antibiotic sensitivity was determined using the disk diffusion method.

### 2.3. Data Acquisition, and Study Variables

Data was collected from the hospital database and patients’ medical records and reviewed by two independent researchers. All inconsistencies between findings were evaluated by a third physician, who reviewed the data. Demographic data (age, sex, age range), clinical history (including cholecystectomy history for Group A), presenting symptoms, ERCP timing, the Tokyo severity score, etiology of obstruction, and duration of illness were recorded. Laboratory data included white blood count, inflammatory tests (CRP), and liver function tests (total bilirubin, number of platelets, and INR). The primary variables of interest were the identification of microbial species in bile samples and their antibiotic resistance patterns as follows: ESBL—extended-spectrum beta-lactamases; MRSA—methicillin-resistant Staphylococcus Aureus; VRE—vancomycin-resistant Enterococci; CRE—carbapenem-resistant Enterobacteriaceae; MDR—multidrug-resistant.

### 2.4. Statistical Analysis

Data analysis was performed using SPSS version 27. Descriptive statistics provided a summary of demographic and clinical characteristics. The proportions of microbial species and resistance patterns between the two groups were compared using the Chi-square test or Fisher’s exact test, based on the frequency assumptions. For continuous data, we calculated the independent samples t-test when comparing two means, and the Mann–Whitney U test to compare two medians. A *p*-value < 0.05 was considered statistically significant.

For hypothesis testing, it was assumed that post-cholecystectomy patients have a higher likelihood of presenting with more complex microbial profiles in their bile cultures, as well as having a higher risk of developing antibiotic resistance, compared to non-cholecystectomy patients. The dependent variable was the complexity of the microbial profile in bile cultures, categorized based on the number of different bacterial species identified (sterile, 1 bacterium, 2 bacteria, ≥3 bacteria). The independent variable of primary interest was the cholecystectomy status. The chosen covariates were age, gender, total bilirubin, CRP levels, and the Tokyo severity score. These variables were included to adjust for potential confounders. Logistic regression models were used to identify factors independently associated with microbial complexity and resistance patterns.

## 3. Results

Table 1 presents the background characteristics of patients, divided into those with and without cholecystectomy. The analysis included a total of 488 patients, with 102 who had a history of cholecystectomy and 386 who did not go through the procedure. Age distribution showed a slightly higher mean age in the cholecystectomy group (68.7 years) compared to the non-cholecystectomy group (66.3 years), but this difference was not statistically significant (*p*-value = 0.062). Gender distribution was fairly balanced in both groups, with males constituting 47.1% of the cholecystectomy group and 53.1% of the non-cholecystectomy group.

The prevalence of symptoms like abdominal pain, jaundice, and fever and chills were similar in both groups, with no significant differences (*p*-values: 0.704, 0.722, and 0.948, respectively). In terms of ERCP (endoscopic retrograde cholangiopancreatography) timing, a comparable distribution was observed across emergent, urgent, and late categories in both groups (*p*-value = 0.755).

The duration of hospital stay was slightly higher in the cholecystectomy group (7.3 days, IQR 4.9) compared to the non-cholecystectomy group (7.1 days, IQR 5.1), but this difference was not statistically significant (*p*-value = 0.543). Tokyo severity scores, categorized into grades I, II, and III, showed a balanced distribution across both groups, with no significant differences observed (*p*-value = 0.379). A higher percentage of malignant causes was observed in the non-cholecystectomy group (52.6%) compared to the cholecystectomy group (42.2%), while benign causes were more common in the cholecystectomy group (57.8%) compared to the non-cholecystectomy group (47.4%), with no statistical significance (*p*-value = 0.060).

C-reactive protein (CRP) levels were significantly higher in the cholecystectomy group (129.5 mg/L) compared to the non-cholecystectomy group (109.1 mg/L), with a *p*-value of 0.032, suggesting a significantly stronger inflammatory response in patients who had a history of cholecystectomy. Total bilirubin levels were also significantly higher among those who had cholecystectomy (7.9 g/dL vs. 6.7 g/dL, *p*-value = 0.041), indicating a potential difference in liver function or biliary obstruction between the two groups.

In terms of bile culture results, a significant difference was observed between the study groups (*p*-value < 0.001). In the cholecystectomy group, 18.6% had sterile cultures, while 33.4% of the non-cholecystectomy group had sterile cultures. The presence of one bacterium was slightly more common in the cholecystectomy group (41.2% vs. 38.3%), whereas 27.5% of cholecystectomy patients had two bacteria compared to 25.1% in the non-cholecystectomy group. Notably, a higher proportion of patients in the cholecystectomy group had cultures with ≥ three bacteria (12.7%) compared to the non-cholecystectomy group (3.1%).

Blood culture results also showed a significant difference between acute cholangitis patients with a previous history of cholecystectomy and those without (*p*-value = 0.005). Sterile cultures were more common in the non-cholecystectomy group (60.0%) compared to the cholecystectomy group (56.0%). In the cholecystectomy group, 36.0% had cultures with one bacterium, compared to 28.8% in the non-cholecystectomy group. Both groups had a low prevalence of cultures with two bacteria and no cases with ≥ three bacteria, as presented in Table 2.

Table 3 presents a comparison in the etiology of obstruction in patients with and without cholecystectomy. The majority of cases in both groups were due to choledocholithiasis, with a notably high prevalence in the cholecystectomy group (96.6% or 57 cases) compared to the non-cholecystectomy group (91.3% or 167 cases). However, this difference was not statistically significant (*p*-value = 0.172). Other benign causes, such as Vaterian ampulloma, benign choledochal stenosis, Mirizzi syndrome, and liver abscess were relatively rare, and their proportions did not significantly differ between the two groups, with *p*-values of 0.199, 0.426, 0.976, and 0.569, respectively.

In terms of malignant obstructions, pancreatic cancer was the most common cause in both groups, with 53.5% (23 cases) in the cholecystectomy group and 55.7% (113 cases) in the non-cholecystectomy group. This similarity was statistically non-significant (*p*-value = 0.794). Other malignant causes, including cholangiocarcinoma, malignant Vaterian ampulloma, malignant extrinsic compression, and gallbladder cancer, were also compared. None of these causes showed a significant difference in prevalence between the cholecystectomy and non-cholecystectomy groups.

A significant finding was the high prevalence of extended-spectrum beta-lactamases (ESBLs)-producing organisms in the cholecystectomy group, accounting for 44.2% (19 cases) of the isolates, compared to 20.2% (41 cases) in the non-cholecystectomy group. This difference was statistically significant (*p*-value < 0.001), indicating a stronger association of ESBLs with patients who underwent cholecystectomy. The presence of methicillin-resistant Staphylococcus Aureus (MRSA) was relatively low in both groups, with no cases in the cholecystectomy group and 1.0% (two cases) in the non-cholecystectomy group. This difference was not statistically significant (*p*-value = 0.513), suggesting that the cholecystectomy status did not significantly impact the occurrence of MRSA in bile cultures.

**Figure 1:**
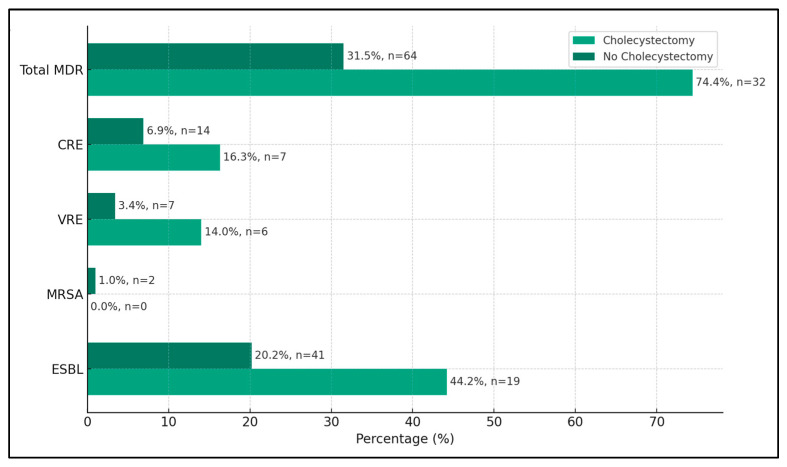
Evidence of multidrug-resistant microorganisms isolated from bile cultures.

Vancomycin-resistant Enterococci (VRE) were more prevalent in the cholecystectomy group, with 14.0% (six cases) compared to 3.4% (7 cases) in the non-cholecystectomy group. This difference was statistically significant (*p*-value = 0.005), suggesting a possible link between cholecystectomy and the presence of VRE. Carbapenem-resistant Enterobacteriaceae (CRE) were also found more frequently in the cholecystectomy group, at 16.3% (seven cases) compared to 6.9% (14 cases) in the non-cholecystectomy group, with a statistically significant difference (*p*-value = 0.046). Overall, the total incidence of multidrug-resistant organisms was markedly higher in the cholecystectomy group at 74.4% (32 cases) compared to 31.5% (64 cases) in the non-cholecystectomy group (*p*-value < 0.001), as seen in Table 4 and Figure 1.

Among the Gram-negative bacteria, *E. coli* was identified significantly more frequently in patients with cholecystectomy (40.2%) compared to those without (28.2%), with a *p*-value of 0.019. *Klebsiella* spp. were found in 15.7% (16 cases) of the cholecystectomy group and 19.2% (74 cases) of the non-cholecystectomy group, but this difference was not statistically significant (*p*-value = 0.419). *Pseudomonas* spp. and *Enterobacter* spp. showed a higher prevalence in the cholecystectomy group, with significant differences (21.6% vs. 2.8% and 13.7% vs. 4.4%, respectively; *p*-values < 0.001). *Acinetobacter* spp. and *Citrobacter* spp. were present in similar proportions in both groups, with no significant differences (*p*-values 0.133 and 0.379, respectively).

For Gram-positive bacteria, *Enterococcus* spp. were significantly more prevalent in the cholecystectomy group (32.4% or 33 cases) compared to the non-cholecystectomy group (21.2% or 82 cases), with a *p*-value of 0.018. *Streptococcus* spp. and *Staphylococcus* spp. were also more commonly found in the cholecystectomy group, with significant differences observed (12.7% vs. 1.6% and 8.8% vs. 2.8%, respectively; *p*-values < 0.001 and 0.007), as seen in Table 5.

Piperacillin/Tazobactam resistance was significantly higher in the cholecystectomy group (23.5%) compared to the non-cholecystectomy group (11.7%), with a *p*-value of 0.002. Resistance to Fluoroquinolones (Ciprofloxacin/Levofloxacin) was observed in 6.9% of the cholecystectomy group, significantly lower than the 16.8% in the non-cholecystectomy group (*p*-value = 0.011). Penems (Meropenem/Imipenem) resistance was significantly higher in the cholecystectomy group (22.5%) than in the non-cholecystectomy group (9.6%), with a *p*-value of less than 0.001.

There was no significant difference in resistance to second generation cephalosporins (*p*-value = 0.473) and third generation cephalosporins (*p*-value = 0.203) between the two groups. However, resistance to fourth generation cephalosporins was significantly different, with 7.8% in the cholecystectomy group compared to 17.9% in the non-cholecystectomy group (*p*-value = 0.013).

Aminoglycoside (Gentamicin/Amikacin) resistance showed no significant difference between the groups (*p*-value = 0.483). Resistance to Ticarcillin/Clavulanic acid was also not significant, with 20.6% in the cholecystectomy group and 11.4% in the non-cholecystectomy group (*p*-value = 0.087). Piperacillin resistance was significantly higher in the cholecystectomy group (56.9%) compared to the non-cholecystectomy group (27.5%), with a *p*-value of less than 0.001, as presented in Table 6.

Table 7 focuses on the development of antibiotic resistance. The odds ratio (OR) for cholecystectomy status was 3.17 (CI = 1.30 to 4.46), indicating that patients with cholecystectomy were more than three times as likely to develop antibiotic resistance compared to those without (*p*-value < 0.001). The total bilirubin levels had an OR of 1.08 (1.02–1.64) and were statistically significant (*p*-value = 0.008), suggesting a modest association with the development of antibiotic resistance. 

**Figure 2:**
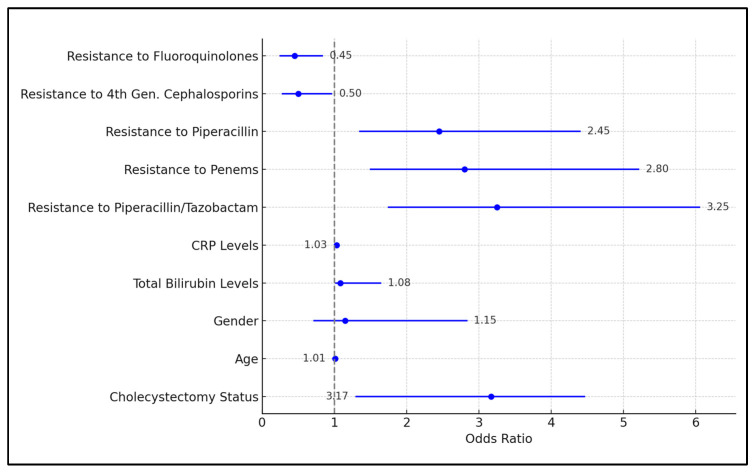
Forest plot analysis for development of antibiotic-resistant bacterial infections.

Resistance to Piperacillin/Tazobactam had an OR of 3.25 (1.75–6.05) with a *p*-value of less than 0.001, indicating a strong association with the development of resistance. Similarly, resistance to Penems (OR = 2.80, 1.50–5.21, *p*-value = 0.001) and Piperacillin (OR = 2.45, 1.35–4.40, *p*-value = 0.003) were significantly associated with increased odds of resistance. Conversely, resistance to fourth generation cephalosporins (OR = 0.50, 0.28–0.96, *p*-value = 0.020) and Fluoroquinolones (OR = 0.45, 0.25–0.83, *p*-value = 0.007) were associated with a reduced likelihood of developing resistance, as presented in Figure 2.

The regression analysis found that cholecystectomy status had a significant association with the development of complex bacterial infections. Patients with cholecystectomy had an odds ratio of 2.45 (CI = 1.56–3.84), indicating they were more than twice as likely to develop complex infections compared to those without cholecystectomy, with a *p*-value of 0.002. Age and gender, with ORs of 1.02 (0.99–1.05) and 1.18 (0.74–1.89) respectively, did not show a significant association with the development of complex bacterial infections.

Total bilirubin levels were found to be significantly associated with the development of complex infections, with an OR of 1.10 (CI = 1.03–1.18, *p*-value = 0.007), suggesting that higher bilirubin levels might be a risk factor for more complex infections. CRP levels also showed a significant association, with an OR of 1.04 (1.01–1.07) and a *p*-value of 0.015, indicating that elevated CRP levels might be predictive of more complex bacterial profiles. However, the Tokyo severity score, a clinical measure used in the management of cholangitis, showed an OR of 1.29 (0.82–2.03) but was not statistically significant in predicting the development of complex infections (*p*-value = 0.273), as seen in Table 8.

## 4. Discussion

### 4.1. Literature Findings

This study provides crucial insights into the comparative analysis of microbial species and multidrug resistance patterns in acute cholangitis patients, particularly emphasizing the effects of prior biliary system manipulation in patients with cholecystectomy. Notably, our findings suggest that such prior interventions may predispose patients to specific and more severe types of infections, possibly linked to the increased usage of antibiotics.

The significant differences observed in our study regarding microbial species between patients with and without cholecystectomy underline the influence of previous biliary system manipulations. Such interventions appear to alter the normal biliary flora, leading to a predominance of certain pathogens. This aligns with the understanding that biliary interventions, including cholecystectomy, can disrupt the native biliary environment, thereby altering susceptibility to specific bacterial infections [28,29].

Our data indicate that patients with a history of cholecystectomy and biliary manipulation are more prone to severe infections. This could be attributed to the altered biliary environment post-surgery, which becomes more conducive to the proliferation of pathogenic bacteria, as seen in the higher prevalence of organisms like Escherichia coli, Klebsiella spp., and Pseudomonas spp. in these patients. These data were confirmed by previous studies [30,31].

Another study’s findings regarding the influence of biliary microbial flora on complications following pancreaticoduodenectomy (PD) align well with our hypotheses and observations regarding bacterial species and the risks of biliary tree manipulation [32]. The high prevalence of positive biliary cultures in 66% of PDs (162 out of 244 cases) underscores the commonality of bacterobilia in such procedures. Notably, the study demonstrates a clear association between specific bacterial species in biliary cultures and postoperative complications. The significant prevalence of polymicrobial cultures, particularly those positive for *Escherichia coli*, *Klebsiella pneumoniae*, and *Enterococcus faecalis*, in surgical site infections (SSIs) and postoperative pancreatic fistula indicates a direct influence of certain bacteria on the type and severity of complications.

Furthermore, the study reveals that monomicrobial *Escherichia coli* bacterobilia is specifically associated with delayed gastric emptying (DGE) as a unique complication, with an odds ratio of 2.94 (1.30–6.70). This finding is particularly intriguing, as it suggests a more nuanced role for individual bacterial species in postoperative outcomes. These results not only validate our hypotheses about the impact of specific biliary microflora on possible outcomes but also provide crucial insights for tailoring postoperative management strategies. 

The microbial spectrum in acute cholangitis has evolved over the years. Initially dominated by *Escherichia coli* and *Klebsiella pneumoniae*, recent studies have reported a diversification in the microbial species involved, including the rise of anaerobic bacteria. A study in rural northern Kyoto, Japan, found that at the first episode of acute cholangitis, the predominant strains belonged to *Escherichia coli* (17.9%), followed by *Klebsiella* spp. (15.5%), whereas at the second episode, *Enterococcus* spp. (35.8%) and *Klebsiella* spp. (27.5%) were more prevalent [33]. This shift in the microbial landscape has important implications for antibiotic therapy.

Multidrug resistance in acute cholangitis is a growing concern. A study analyzing 1764 isolates from positive bile duct cultures in two German tertiary centers found that MDR bacteria were isolated from 24/83 (29%) patients, with biliary stenting identified as an independent risk factor for biliary MDR bacteria [1]. The clinical impact of these MDR organisms is profound, often leading to prolonged hospital stays, increased healthcare costs, and higher mortality rates.

Similarly, a notable finding in our study is the increased prevalence of multidrug-resistant organisms in patients with cholecystectomy. This could be a consequence of heightened antibiotic exposure due to recurrent or severe infections post-biliary manipulation. Studies have highlighted the increasing trend of antibiotic resistance in such settings, necessitating cautious and judicious use of antibiotics [3,34].

A study investigating 321 positive bile cultures from 931 patients with acute cholecystitis who underwent laparoscopic cholecystectomy found that the frequency of enterococci declined while that of *Enterobacteriales*, particularly *E. coli*, increased over time. The incidence of ciprofloxacin-resistant *Enterobacteriales* showed a significant increasing trend, along with the observation of vancomycin-resistant *E. faecium* and carbapenem-resistant *Enterobacteriales* [3]. These findings highlight the changing microbial landscape and resistance patterns in cholecystectomy patients, necessitating a reevaluation of empirical antibiotic therapies.

These findings have important clinical implications. They suggest a need for heightened vigilance in patients with prior biliary manipulations, especially those who have undergone cholecystectomy. Tailoring antibiotic therapy based on individual patient history and potential resistance patterns becomes crucial in this context. Further research should aim to delineate the specific mechanisms by which prior biliary interventions influence microbial profiles and resistance patterns. Longitudinal studies examining the evolution of these patterns in post-cholecystectomy patients could provide deeper insights into effective management strategies.

### 4.2. Study Limitations

This study’s design and methods introduce several limitations, such as being conducted at a single regional hospital in western Romania, and the findings may not be generalizable to other geographic locations or healthcare settings due to potential regional variations in microbial profiles and healthcare practices. Additionally, excluding patients with a history of AC post-ERCP or those on antibiotics for other conditions might have led to a selection bias, potentially influencing the observed microbial profiles and resistance patterns. While the use of advanced diagnostic systems for microbial identification and antibiotic susceptibility testing ensures accuracy, reliance on hospital databases and medical records for data collection may introduce information bias. Moreover, our study did not include data on previous antibiotic use, which is a limitation, as prior antibiotic exposure can significantly influence resistance patterns. This lack of data restricts our ability to directly correlate specific antibiotic treatments with the observed resistance. The VITEK^®^2 system relies on cultures obtained from bile samples, and this method captures only those microbes that are culturable under the specific conditions applied, while the microbial spectrum identified is influenced by factors such as culturing methodologies, conditions, culturability of the microbes, and antibiotic pressure. Lastly, the study’s statistical analysis, though robust, is confined to the data collected during the study period and may not account for long-term trends or emerging microbial strains and resistance mechanisms. 

## 5. Conclusions

This study conclusively demonstrates that acute cholangitis patients with a history of cholecystectomy exhibit significantly different microbial profiles and a higher prevalence of multidrug resistance compared to non-cholecystectomy patients. The cholecystectomy group showed an elevated presence of multidrug-resistant organisms and a higher incidence of specific pathogens like *Escherichia coli* and *Enterococcus* spp. Moreover, the increased resistance to Piperacillin/Tazobactam and Penems in this group suggests a need for reevaluating current antibiotic treatment protocols. These findings highlight the importance of considering surgical history in the management of acute cholangitis to enhance therapeutic effectiveness and patient outcomes. 

## Figures and Tables

**Table 1:** Background characteristics of patients with and without cholecystectomy.

Variables	Cholecystectomy (n = 102)	Non-Cholecystectomy (n = 386)	*p*-Value
Age (mean ± SD)	68.7 ± 10.9	66.3 ± 11.7	0.062
Sex			0.276
Male	48 (47.1%)	205 (53.1%)	
Female	54 (52.9%)	181 (46.9%)	
Age range			0.092
18–39 years	23 (22.5%)	55 (14.2%)	
40–65 years	37 (36.3%)	172 (44.6%)	
>65 years	42 (41.2%)	159 (41.2%)	
Symptoms			
Abdominal pain	79 (77.5%)	292 (75.6%)	0.704
Jaundice	83 (81.4%)	308 (79.8%)	0.722
Fever and chills	29 (28.4%)	111 (28.8%)	0.948
ERCP timing			0.755
Emergent (<48 h)	58 (56.9%)	218 (56.5%)	
Urgent (48–72 h)	24 (23.5%)	81 (21.0%)	
Late (>72 h)	20 (19.6%)	87 (22.5%)	
Duration of hospital stay (median, IQR)	7.3 (4.9)	7.1 (5.1)	0.543
Tokyo severity score			0.379
Grade I	39 (38.2%)	152 (39.4%)	
Grade II	40 (39.2%)	126 (32.6%)	
Grade III	23 (22.5%)	108 (28.0%)	
Etiology of obstruction			0.060
Malignant	43 (42.2%)	203 (52.6%)	
Benign	59 (57.8%)	183 (47.4%)	

SD—standard deviation; IQR—interquartile range.

**Table 2:** Comparison of laboratory data and culture results between patients with and without cholecystectomy.

Variables	Cholecystectomy (n = 102)	Non-Cholecystectomy (n = 386)	*p*-Value
WBC (4.5–11.0 thousands/mm^3^)	12.2 ± 6.0	12.6 ± 5.8	0.538
CRP (0–10 mg/L)	129.5 ± 71.4	109.1 ± 88.3	0.032 *
Total bilirubin (0.3–1.2 g/dL)	7.9 ± 6.2	6.7 ± 5.0	0.041 *
PLT (150–450 thousands/mm^3^)	256.1 ± 94.7	273.8 ± 102.5	0.116
INR (0.8–1.18)	3.2 ± 1.6	2.9 ± 1.7	0.109
Bile cultures (n = 488)	(n = 102)	(n = 386)	<0.001 *
Sterile	19 (18.6%)	129 (33.4%)	
1 bacterium	42 (41.2%)	148 (38.3%)	
2 bacteria	28 (27.5%)	97 (25.1%)	
≥3 bacteria	13 (12.7%)	12 (3.1%)	
Blood cultures (n = 341)	(n = 75)	(n = 266)	0.005 *
Sterile	42 (56.0%)	197 (60.0%)	
1 bacterium	27 (36.0%)	62 (28.8%)	
2 bacteria	6 (8.0%)	7 (11.3%)	
≥3 bacteria	0 (0.0%)	0 (0.0%)	

* Statistically significant at α = 0.05; WBC—white blood cells; CRP—C-reactive protein; PLT—platelets; INR—International Normalized Ratio; a *p*-value threshold of less than 0.05 was set for statistical significance.

**Table 3:** Comparison of etiology of obstruction between patients with and without cholecystectomy.

Etiology of Obstruction	Cholecystectomy (n = 102)	Non-Cholecystectomy (n = 386)	*p*-Value
Benign (n = 242)	(n = 59)	(n = 183)	
Choledocholithiasis	57 (96.6%)	167 (91.3%)	0.172
Vaterian ampulloma	0 (0.0%)	5 (2.7%)	0.199
Benign choledochal stenosis	1 (1.7%)	7 (3.8%)	0.426
Mirizzi syndrome	1 (1.7%)	3 (1.6%)	0.976
Liver abscess	0 (0.0%)	1 (0.5%)	0.569
Malignant (n = 246)	(n = 43)	(n = 203)	
Pancreatic cancer	23 (53.5%)	113 (55.7%)	0.794
Cholangiocarcinoma	10 (23.3%)	57 (28.1%)	0.518
Malignant vaterian ampulloma	6 (14.0%)	25 (12.3%)	0.768
Malignant extrinsic compression	2 (4.7%)	5 (2.5%)	0.433
Gallbladder cancer	2 (4.7%)	3 (1.5%)	0.180

**Table 4:** Evidence of multidrug-resistant microorganisms isolated from bile cultures.

Bile Samples (n = 488)	Cholecystectomy (n = 102)	Non-Cholecystectomy (n = 386)	*p*-Value
Extended-spectrum beta-lactamases = 60 (12.3%)	19 (44.2%)	41 (20.2%)	<0.001 *
Methicillin-resistant Staphylococcus Aureus = 2 (0.4%)	0 (0.0%)	2 (1.0%)	0.513
Vancomycin-resistant Enterococci = 13 (2.7%)	6 (14.0%)	7 (3.4%)	0.005 *
Carbapenem-resistant Enterobacteriaceae = 22 (4.5%)	7 (16.3%)	14 (6.9%)	0.046 *
Total multidrug-resistant = 97 (19.9%)	32 (74.4%)	64 (31.5%)	<0.001 *

* Statistically significant at α = 0.05.

**Table 5:** Comparison microbial profiles from bile samples between patients with and without cholecystectomy.

Microbial Identification	Cholecystectomy (n = 102)	Non-Cholecystectomy (n = 386)	*p*-Value
Gram-negative			
*Escherichia coli* = 150/488 (30.7%)	41 (40.2%)	109 (28.2%)	0.019 *
*Klebsiella* spp. = 90/488 (18.4%)	16 (15.7%)	74 (19.2%)	0.419
*Pseudomonas* spp. = 33/488 (6.7%)	22 (21.6%)	11 (2.8%)	<0.001 *
*Enterobacter* spp. = 31/488 (6.3%)	14 (13.7%)	17 (4.4%)	<0.001 *
*Acinetobacter* spp. = 10/488 (2.1%)	4 (3.9%)	6 (1.6%)	0.133
*Citrobacter* spp. = 17/488 (3.5%)	5 (4.9%)	12 (3.1%)	0.379
Gram-positive			
*Enterococcus* spp. = 115/488 (23.6%)	33 (32.4%)	82 (21.2%)	0.018 *
*Streptococcus* spp. = 19/488 (3.9%)	13 (12.7%)	6 (1.6%)	<0.001 *
*Staphylococcus* spp. = 20/488 (4.1%)	9 (8.8%)	11 (2.8%)	0.007 *

* Statistically significant at α = 0.05.

**Table 6:** Evaluation of antibiotic resistance patterns from bile cultures compared between patients with and without cholecystectomy.

Antibiotic Resistance	Cholecystectomy (n = 102)	Non-Cholecystectomy (n = 386)	*p*-Value
Ampicillin/Sulbactam = 22/66 (33.3%)	7 (6.9%)	15 (3.9%)	0.197
Piperacillin/Tazobactam = 69/344 (20.1%)	24 (23.5%)	45 (11.7%)	0.002 *
Fluoroquinolones (Ciprofloxacin/Levofloxacin = 72/457 (15.8%)	7 (6.9%)	65 (16.8%)	0.011 *
Penems (Meropenem/Imipenem) = 60/339 (17.7%)	23 (22.5%)	37 (9.6%)	<0.001 *
2nd Gen. Cephalosporin = 14/54 (25.9%)	4 (3.9%)	10 (2.6%)	0.473
3rd Gen. Cephalosporin = 16/356 (4.5%)	5 (4.9%)	11 (2.8%)	0.203
4th Gen. Cephalosporin = 77/337 (22.8%)	8 (7.8%)	69 (17.9%)	0.013 *
Aminoglycoside(Gentamicin/Amikacin) = 14/427 (3.3%)	4 (3.9%)	10 (2.6%)	0.483
Ticarcillin/Clavulanic = 65/202 (32.2%)	21 (20.6%)	44 (11.4%)	0.087
Piperacillin = 164/294 (55.8%)	58 (56.9%)	106 (27.5%)	<0.001 *

* Statistically significant at α = 0.05.

**Table 7:** Logistic regression analysis for development of MDR bacterial species.

Variables	Odds Ratio	*p*-Value
Cholecystectomy Status	3.17 (1.30–4.46)	<0.001 *
Age	1.01 (0.98–1.04)	0.476
Gender	1.15 (0.72–2.83)	0.556
Total Bilirubin Levels	1.08 (1.02–1.64)	0.008 *
CRP Levels	1.03 (1.00–1.06)	0.059
Resistance to Piperacillin/Tazobactam	3.25 (1.75–6.05)	<0.001 *
Resistance to Penems	2.80 (1.50–5.21)	0.001 *
Resistance to Piperacillin	2.45 (1.35–4.40)	0.003 *
Resistance to 4th Gen. Cephalosporins	0.50 (0.28–0.96)	0.020 *
Resistance to Fluoroquinolones	0.45 (0.25–0.83)	0.007 *

* Statistically significant at α = 0.05.

**Table 8:** Logistic regression analysis for development of complex bacterial infections.

Variables	Odds Ratio	*p*-Value
Cholecystectomy Status	2.45 (1.56–3.84)	0.002 *
Age	1.02 (0.99–1.05)	0.123
Gender	1.18 (0.74–1.89)	0.489
Total Bilirubin	1.10 (1.03–1.18)	0.007 *
CRP Levels	1.04 (1.01–1.07)	0.015 *
Tokyo Severity Score	1.29 (0.82–2.03)	0.273

CRP—C-reactive protein; * statistically significant at α = 0.05; the complexity of the microbial profile in bile cultures, categorized based on the number of different bacterial species identified (sterile, one bacterium, two bacteria, ≥three bacteria).

## Data Availability

Data available on request.
